# Introduction to the Special Issue on Pediatric Acute Myeloid Leukemia: Current Management and Future Directions

**DOI:** 10.3390/children8080698

**Published:** 2021-08-12

**Authors:** Alexandra M. Stevens, Michele S. Redell

**Affiliations:** Texas Children’s Cancer and Hematology Centers, Baylor College of Medicine, Houston, TX 77030, USA; amsteven@texaschildrens.org

This Special Issue brings together an original research report, a fascinating case report, and three timely reviews on a variety of topics related to pediatric leukemia. The original research report by Dr. Tremolada and colleagues [1] addresses the important question of how a child’s experiences as a cancer patient influence developmental progress and social competencies. The authors compared social and verbal skills for preschool-aged survivors of acute lymphoblastic leukemia (ALL) with those of age-matched controls. They found that the ALL survivors performed less well in multiple domains, most notably in adaptive functioning. The fact that measurable delays could be identified for these patients suggests that similar delays are likely for young children with AML too. The results could inform future “social therapy” programs for children facing long hospitalizations.

The case report by Prudowsky et al. [2] describes the intriguing case of a newborn without phenotypic signs of Down syndrome, but with an elevated WBC and megakaryoblasts in the peripheral blood. Before initiating very aggressive chemotherapy for acute megakaryoblastic leukemia (AMKL), the astute clinical team evaluated the baby for mosaic trisomy 21. Indeed, the baby began to recover on her own, and molecular testing ultimately confirmed the case of transient abnormal myelopoiesis (TAM), also known as transient myeloproliferative disorder (TMD), in a newborn with mosaic trisomy 21. This case highlights the value of collaborating with molecular pathology colleagues and rapidly implementing advanced targeted testing to spare this infant unnecessary and toxic chemotherapy.

This Special Issue also brings us three outstanding reviews on pertinent and rapidly evolving issues in pediatric AML therapies. The review by Epperly and colleagues [3] from St. Jude’s addresses the new and promising field of T cell immunotherapies for AML. While new agents such as bispecific T cell engager (BiTE) antibodies and chimeric antigen receptor (CAR) T cells represent significant advances for children with ALL, for AML, finding suitable targets that are specific for myeloblasts without excessive toxicity to normal hematopoietic cells has proved more challenging. This review covers the current state of the effort and provides a glimpse at a hopeful future for AML immunotherapies.

The review by Chen and Glasser [4] provides a status report on the many other kinds of targeted therapies in development for pediatric AML. Small molecule inhibitors of FLT3 have become the standard of care for children with AML with FLT3/ITD mutations. Newer inhibitors targeting signaling pathways, epigenetic factors, and microenvironment interactions are in various stages of development. Antibody–drug conjugates (ADC) are another class of targeted immunotherapies, but unlike BiTE antibodies, ADCs do not require T cell activity. The relative success of gemtuzumab ozogamicin has led to an explosion of interest in developing new ADCs, as discussed in this review.

Finally, the review by Conneely and Stevens [5] describes the remarkable success story of acute promyelocytic leukemia (APL). Driven by basic science and internationally collaborative clinical research, cure rates for this uncommon subtype of AML now rival those for low-risk pre-B ALL and Wilms tumor. The editors are grateful to all the authors who contributed papers for this Special Issue.

## Data Availability

Not applicable.
